# Hypoxia-Driven Mechanisms of Drug Resistance in Prostate Cancer

**DOI:** 10.3390/cancers18060899

**Published:** 2026-03-11

**Authors:** Madeline R. Ressel, Caitlyn E. Flores, Noel A. Warfel

**Affiliations:** 1 Department of Urology, The University of Arizona, Tucson, AZ 85721, USA; 2 Cancer Biology Graduate Interdisciplinary Program, University of Arizona Cancer Center, Tucson, AZ 85724, USA; 3 Department of Cellular & Molecular Medicine, The University of Arizona, Tucson, AZ 85721, USA; 4 Department of Biochemistry and Molecular Biology, The Medical University of South Carolina, Charleston, SC 29425, USA

**Keywords:** prostate cancer, hypoxia, therapeutic resistance, CRPC, bone metastasis

## Abstract

Prostate cancer is the most common non-cutaneous malignancy in men and is the second leading cause of male cancer-related mortality. Unlike many cancers, prostate cancer lacks clear genetic driver mutations, suggesting that factors in the tumor microenvironment contribute to the genesis and progression of this disease. Hypoxia, or a physiological state of low oxygen, is a universal characteristic of solid tumors that enhances disease progression and therapeutic resistance. Prostate cancer develops in a hypoxic microenvironment and primarily metastasizes to bone, where oxygen availability is similarly limited. Therefore, hypoxia is a major obstacle to the effective treatment of prostate cancer across all disease stages. Clinically, hypoxia is correlated with worse patient outcomes, largely because it drives resistance to the frontline therapies used to treat both primary and metastatic prostate cancer. Despite the established role of hypoxia in prostate cancer progression and drug resistance, it has not been successfully targeted therapeutically. Emerging evidence indicates that exposure to distinct temporal patterns of hypoxia (acute, cyclic, and chronic) elicits unique cellular adaptations that dictate tumor growth and survival. This review synthesizes current evidence regarding the role of hypoxia in promoting resistance to therapy in prostate cancer.

## Simple Summary

Hypoxia, or low oxygen, is a hallmark of solid tumor biology that is associated with drug resistance and poor prognosis. This is particularly true for prostate cancer, which is exposed to hypoxia at inception and throughout the course of disease progression. Despite the established role of hypoxia as a major mediator of drug resistance in prostate cancer, it has not been successfully targeted therapeutically. Emerging evidence indicates that exposure to distinct temporal patterns of hypoxia (acute, cyclic, and chronic) elicits unique cellular adaptations that dictate tumor growth and survival. This review provides an overview of the mechanisms that drive hypoxia-mediated drug resistance in prostate cancer.

## 1. Introduction

### 1.1. Prostate Cancer

Prostate cancer (PCa) is the most common cancer in men. With 313,780 cases expected in 2025 alone, it is responsible for 30% of all male cancer diagnoses. Of these cases, 11.4% are predicted to be fatal, making PCa a leading cause of cancer deaths, second only to lung cancer [1]. Despite a favorable 5-year survival rate (>98%) for patients diagnosed with localized disease, a subset (10–20%) of these patients will progress to advanced disease [2]. Progression to metastatic disease, independent of hormone status, is the leading cause of PCa mortality, reducing the 5-year survival rate to 37% [1].

Age is the primary risk factor for PCa [3]. Unlike most cancers, PCa infrequently harbors point mutations, but instead undergoes extensive chromosomal rearrangements that serve as oncogenic drivers of PCa [4,5]. Localized PCa exhibits spatial and genetic heterogeneity, and lineage tracing studies indicate that metastatic lesions can be traced back to primary tumors, implying that aggressive tumor cell populations arise early during tumorigenesis [4,6,7]. Consequently, age-associated changes in the tumor microenvironment (TME) may play a critical role in disease initiation and progression. Beyond the prostate epithelium, the surrounding stroma also undergoes significant age-related changes, including genetic alterations, progenitor cell aging, increased reactive oxygen species (ROS), altered androgen receptor activity, and hypoxia [8,9]. Hypoxia, a physiological state of low oxygen, arises naturally in the aging prostate and often precedes tumor development. Clinical BOLD MRI evidence demonstrates that prostate tumors preferentially emerge in the hypoxic peripheral zone, where oxygen limitation is attributable to age-associated perfusion deficits and tissue atrophy, rather than elevated proliferative burden [10,11]. In addition, ~84% of PCa patients with advanced disease will develop bone metastases, which are characteristically hypoxic [12,13]. Together, low-oxygen conditions in the peripheral zone and within primary and metastatic tumors highlight hypoxia as a defining feature of PCa.

### 1.2. Hypoxia

Hypoxia is a hallmark of solid tumor biology. In PCa and other malignancies, hypoxia is broadly associated with poor patient outcomes by suppressing anti-tumor immunity, promoting cell migration and invasion, enhancing cell survival, and driving therapeutic resistance [14]. The cellular response to hypoxia is largely driven by transcriptional changes downstream of hypoxia-inducible factors (HIFs), a family of transcription factors that are stabilized under low-oxygen conditions. HIF-1α and HIF-2α are the most well studied isoforms, and when activated, they stimulate the expression of hundreds of genes responsible for regulating the cellular response to hypoxia. In normoxia, HIF-1α is hydroxylated via oxygen-dependent prolyl hydroxylases (PHDs) and marked for proteasomal degradation by Von-Hippel-Lindau (VHL) E3 ubiquitin ligase. In hypoxia, HIF-1α is stabilized and translocates to the nucleus, where it dimerizes with HIF-1β and binds hypoxia response elements (HREs) to activate transcription of target genes [15,16]. While the field has largely attributed adaptation to hypoxia to HIF-mediated transcriptional changes, several HIF-independent pathways have also been described, including PIM kinases, NF-κB, p53, mTOR, and c-myc, and many of these pathways experience crosstalk with HIFs [17]. Together, these changes allow tumor cells to adapt and thrive in a hypoxic TME.

### 1.3. Acute, Cyclic, and Chronic Hypoxia

Hypoxia can be categorized into three temporal subtypes: acute, cyclic, and chronic. Acute hypoxia is a transient, perfusion-limited event caused by a temporary pause in blood flow. Cyclic, or intermittent, hypoxia involves repeated interruptions of blood flow, resulting in cycles of acute hypoxia followed by reoxygenation. Chronic, or diffusion-limited, hypoxia is a prolonged state that arises from spatial separation between cells and the vasculature. While both acute and cyclic hypoxia are reversible upon restoration of blood flow and oxygen supply, chronic hypoxia induces more permanent cellular changes [14,17]. These temporal hypoxia patterns drive distinct biological outcomes. The response to acute hypoxia is well characterized, marked by induction of canonical HIF target genes such as glucose transporters (GLUT1), pro-angiogenic factors (VEGF1), and pH regulators (CAIX) [18]. In cyclic hypoxia, repeated fluctuations between hypoxia and reoxygenation generate high levels of ROS [19]. Elevated ROS promotes genomic instability and directly modulates signaling pathways that regulate proliferation and survival, including MAPK, PI3K/Akt, and NF-κB. In contrast to the reversible nature of acute and cyclic hypoxia, chronic hypoxia imposes persistent selective pressure [20,21]. While some cells adapt, others succumb to prolonged oxygen deprivation, giving rise to the classical necrotic core characteristic of solid tumors. Chronic hypoxia is the expected outcome in the context of tissue atrophy, as is observed with age in the peripheral zone of the prostate [10]. Despite the well-established presence of hypoxia in PCa, exposure to different temporal subtypes of hypoxia is emerging as a key factor that dictates the sensitivity of tumor cells to anti-cancer therapies. This review will synthesize current evidence on hypoxia in PCa, highlighting its role in shaping tumor biology across both primary and metastatic sites, and examining how these adaptations contribute to therapeutic resistance.

## 2. Localized Disease

**Figure 1:**
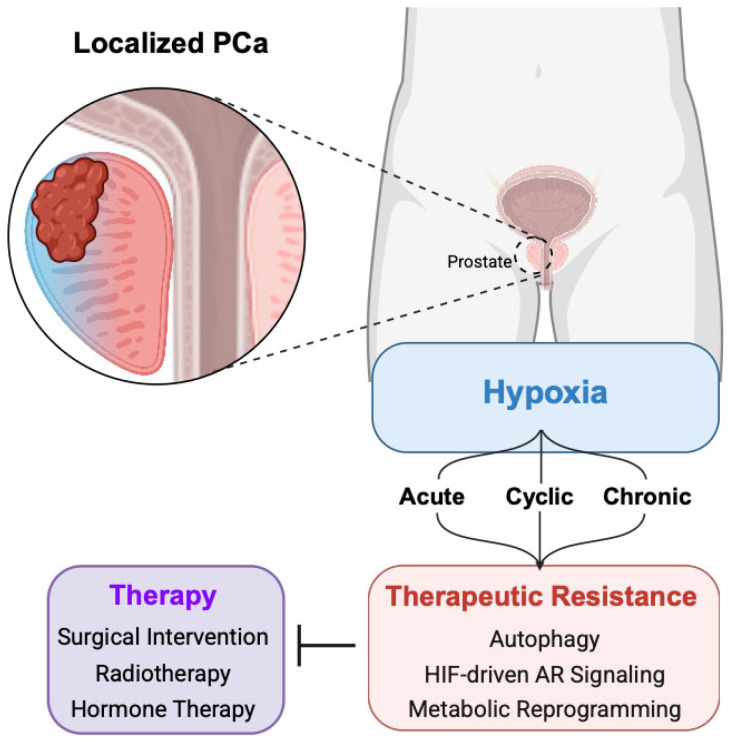
Treatment modalities for localized PCa and hypoxia-induced mechanisms of resistance. Created in BioRender. Warfel, N. https://BioRender.com/ww55n86 (accessed on 3 March 2026).

Patients with localized PCa generally have a favorable prognosis. However, a subset will experience biochemical recurrence and disease progression despite definitive therapy. Treatment selection remains challenging due to heterogeneity in recurrence risk and imperfect stratification tools. In the following sections, we discuss how tumor hypoxia intersects with standard-of-care approaches for localized PCa, including surgery, radiotherapy, and hormone therapy, to promote therapeutic resistance and disease progression (Figure 1).

### 2.1. Surgical Resection and Recurrence

Radical prostatectomy (RP) is the standard surgical treatment for patients with clinically localized PCa. RP includes the removal of the prostate, surrounding tissue, and seminal vesicles. Nearby lymph nodes may also be removed during pelvic lymphadenectomy. Patient candidacy is assessed by age, Gleason Score, PSA levels, and the absence of metastatic disease [22]. In a study of 11,864 men with PCa, 53% underwent RP [23]. Biochemical recurrence (BCR) following RP remains a clinical challenge, impacting 20–40% of patients [24,25]. BCR is defined as two consecutive PSA values of >0.2 ng/mL following RP and significantly increases the risk of metastasis and mortality among patients with poor prognostic features [26,27,28]. However, assessing individual patient risk for BCR has proven to be complex. Current clinical indicators of BCR include Gleason Score, tumor stage, positive surgical margins, and serum PSA levels/kinetics [26,29,30]. However, none of these metrics are highly accurate predictors of patients with indolent vs. aggressive disease, which reflects the intrinsic heterogeneity of disease and patient populations, demonstrating the need for additional identifiers for BCR. Several studies have evaluated hypoxia as a predictive marker for BCR [31,32,33,34,35,36,37]. A 2008 study following a cohort of 289 RP patients demonstrated that increased histological staining of hypoxia markers, VEGFR, HIF-1α, and osteopontin, was associated with a shorter time to BCR, independent of tumor stage, Gleason score, serum PSA levels, and margin status [33]. Building upon these findings, another group generated a 28-gene hypoxia signature that demonstrated value as an independent predictive factor of BCR in 11 RP patient cohorts (*n* = 48–491) [31]. However, subsequent analyses revealed that this signature lacked predictive value in a cohort of high-risk PCa patients [32]. Similarly, a 2014 study assessing three distinct hypoxia RNA signatures (26, 51, and 99 genes) in two surgical cohorts (*n* = 108, *n* = 110) found that the chosen hypoxia genes did not provide significant predictive value alone [34,35,36]. Instead, a multi-variate analysis consisting of markers of hypoxia and genomic instability provided a strong association with BCR [37]. There are multiple considerations that could account for the inconsistency of using hypoxia as a biomarker in these reports. The existing hypoxia gene signatures are commonly derived from canonical HIF-1α downstream targets, which predominantly reflect early or acute hypoxia-associated transcriptional responses. Conversely, genomic instability, which is strongly correlated with aggressive PCa, is more closely associated with chronic hypoxia, where sustained oxygen deprivation impairs DNA repair mechanisms and promotes mutational accumulation [20,38]. Collectively, hypoxia-based biomarkers show promise for BCR risk stratification following RP, and incorporating temporal hypoxia subtypes may improve their predictive value across clinical cohorts.

### 2.2. Radiation

Radiation therapy is a standard frontline treatment for PCa patients; the most common modalities include internal radiation therapy (14% of patients) and external beam radiation therapy (12% of patients) [23]. Like radical prostatectomy, BCR following primary radiotherapy poses a significant clinical challenge. In this context, BCR is defined as a rise in serum PSA +2.0 ng/mL above the post-treatment nadir, in contrast to >0.2 ng/mL following prostatectomy. In both groups, BCR is the strongest, albeit limited, predictor of recurrent disease, with PSA kinetics and pre-treatment Gleason score as key measures for risk stratification.

In addition to its use as a primary treatment modality, radiotherapy is employed as both an adjuvant and salvage therapy. Early studies suggested that adjuvant radiotherapy post-prostatectomy reduces the risk of BCR [28,39,40]. However, subsequent studies demonstrated that adjuvant radiotherapy is associated with increased adverse events and does not confer significant improvement in metastasis-free survival [39,40,41,42]. Further, treating patients with salvage radiotherapy post-BCR resulted in fewer adverse events and prevented the over-treatment of patients who did not experience recurrence. One study proposed that salvage radiotherapy provides improved metastasis-free survival compared to adjuvant radiotherapy [43]. Based on these considerations, salvage radiotherapy is recognized as the current standard of care following prostatectomy, though the optimal timing of intervention remains an active discussion [41,42,44,45].

#### 2.2.1. Oxygen Fixation

The effect of hypoxia on the efficacy of radiotherapy is well established and can be traced back decades [46]. Radiotherapy is ~2.5–3× more effective in well-oxygenated tumors than in hypoxic tumors [47]. This oxygen-dependent effect arises because ionizing radiation generates ROS via water radiolysis, producing oxidative DNA damage and single- and double-strand breaks that compromise clonogenic replication [48]. The increased efficacy of radiotherapy in oxygenated tumors is described by the oxygen fixation hypothesis, which proposes that radiation-generated DNA radicals interact with oxygen, resulting in irreparable damage, whereas in hypoxia, DNA radicals will be restored to their original condition [49]. Notably, DNA repair pathways and protein expression in hypoxia are both time- and concentration-dependent (reviewed in Section 3.2). Consequently, error-prone repair of radiation-induced DNA damage in hypoxia can enhance genomic instability, further promoting therapeutic resistance and aggressive disease [50].

#### 2.2.2. Radiation-Induced Hypoxia

Beyond desensitizing PCa cells to radiation prior to treatment, radiation therapy can also induce tumor hypoxia by damaging endothelial cells [51,52,53]. Ionizing radiation induces endothelial cell apoptosis, vascular rarefaction, and impaired perfusion, leading to transient or sustained reductions in oxygen delivery [51,53]. Consequently, this stimulates angiogenic signaling through HIF-mediated transcription of vascular endothelial growth factor (VEGF), among other survival mechanisms, including enhanced DNA repair, antioxidant response, and modulation of survival kinases [51]. Alongside classical angiogenesis, it has been reported that irradiated tumors recruit pro-angiogenic bone marrow–derived CD11b^+^ myeloid cells via HIF-1–dependent upregulation of SDF-1/CXCL12. This vasculogenic response enables vascular recovery and supports tumor recurrence following radiation [52]. Beyond tumor cells, radiation exposure was demonstrated to induce LDHA expression and increase lactate production in fibroblasts, resulting in extracellular acidification and fibroblast-to-myofibroblast differentiation via pH-dependent TGF-β signaling [54]. Notably, increased lactate is associated with an immunosuppressive TME and progression to castration-resistance in PCa [54,55,56]. Given these reciprocal interactions, hypoxia acts not only as a pre-existing barrier to radiation efficacy but also drives adaptive resistance.

#### 2.2.3. Autophagy

Another mechanism by which hypoxia modulates radioresistance is autophagy, a lysosome-mediated degradation pathway that enables cells to recycle damaged proteins and organelles under metabolic stress. Autophagy can limit radiotherapy efficacy through multiple mechanisms, including enhanced DNA damage repair, reduced intracellular ROS, and activation of pro-survival signaling pathways [57].

Multiple studies have evaluated how hypoxia regulates autophagy-related (ATG) genes in PCa and how autophagy influences PCa radiosensitivity [58,59,60]. A 2019 study reported that the ATG5 promoter region contains at least one HRE sequence in the promoter region, revealing that HIF-1α binds this site to promote ATG5 transcription. LC3-II/I levels were also elevated under acute hypoxia (1% O_2_ for 24 h), indicating increased autophagy relative to normoxia [58]. A 2021 study reported that radioresistant PCa cells (LNCaP, DU145, PC3) exhibit elevated glutamine uptake to maintain redox homeostasis and energy metabolism via HIF-1-mediated metabolic adaptation [60,61,62]. Under glutamine starvation, radioresistant cells engaged ATG5-mediated autophagy to protect against radiation-induced damage. Combined glutamine depletion and autophagy inhibition enhanced radiosensitization. These studies highlight the convergence of hypoxia and autophagy to promote cell survival under stress and suggest that shared adaptations between acute hypoxia and radioresistant states may be leveraged to improve radiotherapy efficacy.

The relationship between hypoxia and autophagy may depend on the extent and duration of hypoxia. A recent study using repeated cyclic hypoxia exposures (multiple cycles ranging from 12 to 48 h+ exposure) reported that intermittent low oxygen exposure suppressed autophagy while promoting 3βHSD1-dependent androgen synthesis and PCa progression [59,63]. Notably, these effects were not evaluated in the context of radiotherapy, emphasizing the need to further investigate the effect of chronic and cyclic hypoxia on sensitivity to radiotherapy. These findings suggest that temporal hypoxia subtypes can differentially regulate autophagy and may also favor distinct survival programs in a context-dependent manner.

### 2.3. Hormone Therapy

#### 2.3.1. ADT and AR Antagonists

Androgens drive PCa progression through activation of the androgen receptor (AR). Accordingly, hormone therapy (HT) remains a cornerstone of PCa management [64,65]. The most common form is androgen deprivation therapy (ADT), which lowers serum testosterone to castrate levels (<50 ng/dL) by suppressing androgen synthesis via LHRH agonists (e.g., Leuprolide, Histrelin) or antagonists (e.g., Degarelix, Abarelix) [65]. In addition, AR antagonists (e.g., Bicalutamide, Enzalutamide) inhibit AR activation through competitive binding of the AR ligand binding domain [66]. Clinically, ADT serves as first-line therapy for metastatic castration-sensitive PCa (mCSPC) and for patients with advanced or high-risk localized disease, where it effectively reduces tumor burden. ADT is also used in the salvage setting after BCR following prostatectomy or radiation therapy [67,68]. Notably, therapeutic efficacy is improved when ADT is combined with AR antagonists and/or chemotherapy [69,70].

#### 2.3.2. Molecular Features of CRPC

Castration-sensitive prostate cancer (CSPC) refers to tumors whose growth remains dependent on AR signaling. Although most patients initially respond to HT, nearly all eventually progress to castration-resistant prostate cancer (CRPC), characterized by tumor growth despite castrate testosterone levels [71,72]. CRPC is highly resistant to all forms of therapy, with the median survival following CRPC diagnosis being only 14 months [13,73]. Multiple mechanisms contribute to HT resistance, most commonly the expression of AR splice variants [74,75], ligand-independent AR activation [76,77], alterations of the tumor microenvironment [78], or the activation of compensatory signaling pathways that sustain growth and survival (e.g., mTOR/PI3K pathways) [79]. Regardless of the mechanisms acquired to evade HT, CRPC cells display activation of EMT programs [80], acquisition of stem-like features [81], and lineage plasticity that often leads to neuroendocrine differentiation, which is a highly aggressive and drug-resistant form of PCa (Reviewed in [82]).

#### 2.3.3. HIF/AR Axis

Hypoxia acts on multiple components of the androgen/AR signaling axis to engage these resistance mechanisms and drive CRPC progression. HIFs have been implicated in disease progression through both AR-dependent and AR-independent mechanisms [83,84,85]. HIF-1α directly enhances AR transcription and interacts with AR as a co-regulator at specific loci, including the PSA promoter [84,85]. Additionally, repeated cycles of acute hypoxia (1% O_2_ for 12–36 h) reportedly promote androgen synthesis through HIF-2α–dependent stabilization of 3βHSD1 [63]. Acting downstream of HIF signaling, the histone demethylase PHF8 promotes CRPC through epigenetic activation of neuroendocrine differentiation genes while also functioning as a chromatin-based co-activator of AR [86,87]. Consistent with these mechanistic observations, several studies report that inhibiting HIF signaling increases sensitivity to HT in CRPC models [83,88], whereas enforced HIF activity has been shown to restore tumor growth under castrate conditions [86]. Together, these findings suggest that concurrent modulation of AR and HIF pathways warrants further investigation.

#### 2.3.4. AR and Hypoxia-Driven Survival Signaling

Hypoxia activates multiple survival pathways implicated in CRPC progression, including PI3K/AKT [79,89], p38/MAPK/Hsp27 [90,91,92], and PIM kinases [76]. In PTEN-deficient tumors, basal hyperactivation of PI3K signaling contributes to a castration-resistant phenotype through activation of Akt and suppression of AR [79,89,93]. However, reciprocal feedback between these pathways complicates therapeutic targeting: AR inhibition can activate PI3K signaling, irrespective of PTEN status, and PI3K inhibition can enhance AR-driven growth and survival [79]. In hypoxia, HIF-1 further amplifies PI3K pathway activation and downstream signaling to molecules that drive cell survival, proliferation, and metabolism [79,93,94]. Subsequently, PI3K inhibition has been tested extensively in prostate cancer and is discussed further in Section 3.2. Differential activation of P38-mitogen-activated protein kinase (MAPK) and heat shock protein 27 (Hsp27) has been implicated in androgen-independent PCa progression, both individually [92,95] and in tandem [90]. Mechanistically, it was reported that p38-mediated Hsp27 activation facilitates AR nuclear translocation in an androgen-depleted environment, promoting survival in both hormone-sensitive [90,95] and neuroendocrine PCa models [91]. Hypoxia exacerbates the p38/Hsp27/AR signaling axis by activating Hsp27 independently of p38, suggesting the presence of additional survival mechanisms [90]. Activation of PIM1 stabilizes AR via direct phosphorylation of residues (e.g., Thr850) that prevent proteasomal degradation, reinforcing AR signaling [76]. PIM is also implicated in several other survival mechanisms [96,97,98,99]. Thus, PIM inhibition has been studied extensively in PCa and is reviewed in Section 3.2.

#### 2.3.5. Metabolic Reprogramming

Hypoxia-driven metabolic reprogramming drives HT resistance [21,100]. In 2017, Geng et al. reported that androgen-sensitive LNCaP cells cultured in acute cycles of hypoxia (1% O_2_ for 24–48 h), but not normoxia, exhibited continued growth despite enzalutamide treatment or AR knockdown. Interestingly, transcriptomic analysis revealed that hypoxia relieved AR-mediated repression of glucose-6-phosphate isomerase (GPI), promoting a transition from AR-dependent pentose phosphate pathway flux to a glycolytic phenotype that supported cell survival [100]. More recently, Cameron et al. [21] demonstrated that chronic hypoxia (1% O_2_ for 144 h) also enabled androgen-sensitive PCa lines (LNCaP, VCaP) to maintain growth following androgen depletion, mimicking the behavior of the castration-resistant LNCaP-V16A line. Shared transcriptional changes between chronic hypoxia and androgen insensitivity revealed glycolysis-associated transcription factor ZNF560 and the glucose transporter, GLUT1, as critical mediators of survival in hypoxia. Importantly, ZNF560 was identified as a significant prognostic factor in patient cohorts, further supporting the clinical relevance of chronic hypoxia–driven metabolic adaptation [21].

Finally, recent evidence suggests that enhanced glycolytic metabolism in stromal cells promotes castration-resistance in PCa [56]. Single-cell transcriptomic analysis of HT-naïve and HT-resistant patient samples revealed that APCDD1^+^ cancer-associated fibroblasts serve as a major source of lactate secretion in a HIF-1-dependent manner following HT. Subsequently, lactate uptake by tumor cells induced expression of AR-V7 through lactylation of the spliceosome component SNRPA, which drives AR alternative splicing and HT resistance. Importantly, blocking lactate transport through MCT inhibition sensitized PCa cells (C4-2, 22RV1) to HT [56]. These results reinforce the importance of lactate metabolism in PCa survival and disease aggression, as demonstrated by prior studies and highlight its potential as a therapeutic target [56,101]. Together, these studies demonstrate that metabolic plasticity, whether driven by hypoxia-induced glycolysis or stromal-derived lactate signaling, promotes a castration-resistant state.

## 3. Metastatic Disease

**Figure 2:**
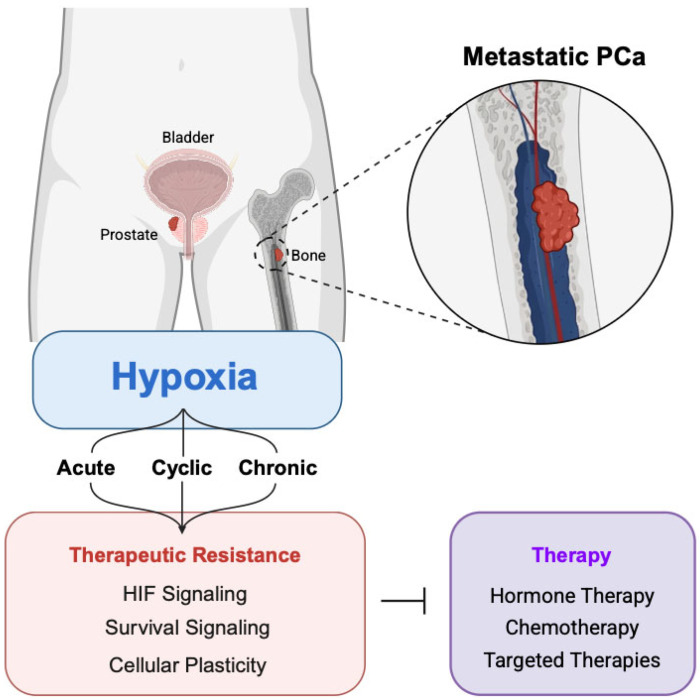
Treatment modalities for metastatic PCa and hypoxia-induced mechanisms of resistance. Created in BioRender. Warfel, N. https://BioRender.com/ld0lxqj (accessed on 8 March 2026).

As discussed in prior sections, definitive local therapies (surgery, radiotherapy) [22,23], and hormone-based treatments (ADT, AR antagonists) [65,69] provide effective disease control for most patients with localized PCa. In contrast, advanced PCa remains a significant clinical challenge. Sustained therapeutic pressure selects for tumors capable of growth despite castrate androgen levels (CRPC). CRPC often coincides with dissemination to distant sites, resulting in metastatic castration-resistant prostate cancer (mCRPC). Notably, metastasis can also occur in androgen-sensitive PCa, defined as metastatic castrate sensitive disease (mCSPC). Independent of hormone status, metastatic disease remains the leading cause of prostate cancer–related mortality [1]. Metastatic lesions most commonly occur in bone, a microenvironment characterized by limited oxygen availability and extreme metabolic constraints [12,13]. Hypoxia signaling has been implicated in PCa bone tropism, in part through upregulating adhesion, homing, and EMT-associated programs, including altered expression of integrins (α6β1, αvβ3) [102], chemokine signaling (CXCR4/CXCL12) [52], and PIM kinases [98]. While bone is the primary site of PCa metastasis, PCa also metastasizes to the lymph nodes, liver, and lungs, and less commonly, the brain, kidneys, and adrenal glands [12]. Once PCa has invaded beyond the primary site, the treatment paradigms shift toward systemic approaches, including cytotoxic chemotherapy, hormone therapy, targeted kinase inhibitors, and immunotherapeutic strategies [103]. However, these interventions are rarely curative. In the sections that follow, we discuss the role of hypoxia as a driver of therapeutic resistance in metastatic PCa (Figure 2).

### 3.1. Taxanes and Genotoxic Agents

Taxanes, namely docetaxel and cabazitaxel, are the most prominently used form of chemotherapy in patients with metastatic PCa (mCSPC and mCRPC), demonstrating improved survival outcomes in combination with hormone therapies [104,105]. Of note, cabazitaxel is a second-generation taxane that is commonly used following progression on docetaxel. Mechanistically, taxanes induce cell death by disrupting microtubule dynamics through binding of the β-subunit of the tubulin heterodimer, promoting collapse of the microtubule’s dynamic-instability state through polymerization. Failure to organize centrosome spindles halts mitosis and leads to apoptosis [106].

Alongside taxanes, genotoxic agents, such as carboplatin, cisplatin (both platinum-based alkylating-like agents), cyclophosphamide (an alkylating agent), and doxorubicin (an anthracycline), are commonly used chemotherapies used to treat mCRPC patients [105]. In contrast to taxanes, alkylating/alkylating-like agents and anthracyclines both induce cell death by inhibiting DNA replication through the formation of DNA adducts and blocking topoisomerase II, respectively [107].

#### 3.1.1. Microtubule Dynamics

Hypoxia is an established mediator of resistance to docetaxel and other taxanes in PCa [108]. Because taxanes preferentially kill cells undergoing mitosis, hypoxic tumor regions can intrinsically reduce treatment efficacy through numerous mechanisms, including context-dependent suppression of mitotic progression, limited drug diffusion in poorly vascularized regions, and activation of stress-adaptive survival programs that support persistence under chronic oxygen deprivation [20,109,110]. Together, these features of the hypoxic TME provide a permissive context for taxane tolerance and the emergence of more therapy-resistant subpopulations.

Alongside altered mitotic activity, microtubule isoforms contribute to taxane resistance. Specifically, increased expression of TUBB3 is associated with poor prognosis for mCRPC and was predictive of docetaxel response in a cohort of 73 mCRPC patients [111]. In line with this, knockdown of TUBB3 in docetaxel-resistant PCa lines (DU145-DR and C4-2-DR) restored sensitivity in vitro [112]. In ovarian cancer cells (A2780), HIF-1α binds to the 3′ promoter region of TUBB3 to promote its expression under anoxia (0% O_2_ for 24–72 h) [113]. Alternatively, in glioblastoma cells (U87MG, GL15), it was shown that HIF-2α, not HIF-1α, regulates TUBB3 expression under acute hypoxia (1% O_2_ for 16–24 h) [114]. Notably, there is evidence to suggest that both tissue type differences and duration of hypoxic exposure influence HIF isoform preference [115]. Together, these results highlight the necessity of differentiating between the duration of hypoxia to gain a clearer understanding of microtubule regulation in hypoxia.

#### 3.1.2. Oxidative Stress

In contrast to taxanes, genotoxic agents promote cell death through DNA damage and the induction of ROS [116]. The interplay between hypoxia signaling and ROS is complex. Hypoxia inherently leads to an increase in cellular ROS production due to disruption of the mitochondrial electron transport chain. Consequently, the metabolic switch to anaerobic glycolysis rather than oxidative phosphorylation in hypoxia is favored [21]. Accordingly, HIF-mediated upregulation of cellular detoxification systems works in a coordinated manner with the antioxidant transcription factor, nuclear factor erythroid 2-related factor 2 (NRF2), to combat oxidative stress [117]. Both HIF and NRF2 stimulate the activity of glutathione-S-transferases (GST), which are required for reduced glutathione (GSH) to directly conjugate ROS and xenobiotics (i.e., chemotherapeutics). Simultaneously, NRF2 activation directly increases the expression of antioxidant genes, including NADPH oxidoreductase-1 (NQO1), superoxide dismutases (SOD), and glutathione peroxidases (GPx) [117,118]. Given the centralized role of mitochondria in cellular ROS production, mitophagy provides an additional layer of protection against hypoxia- and chemotherapy-induced oxidative stress. Mitophagy describes a specialized form of autophagy that clears damaged/dysfunctional mitochondria, which reduces cellular ROS and apoptotic signaling [119]. Specifically, hypoxia promotes receptor-mediated mitophagy through HIF-mediated transcription of BNIP3 and BNIP3L, which directly bind LC3II to facilitate autophagosome clearance of mitochondria. Increased mitophagy has been implicated in cisplatin resistance across multiple malignancies, including ovarian, lung, and breast cancer [120]. Enhanced mitophagy in CRPC cells is reported to promote resistance to docetaxel [121] and more recently, enzalutamide [122].

Paradoxically, ROS can also enhance tumor cell survival in hypoxia by functioning as a signaling intermediate that stabilizes HIF activity and activates stress-adaptive pathways. For example, both HIF and ROS stimulate expression of drug efflux pumps (i.e., ABCB1/MDR1) through direct transcriptional upregulation and ROS-responsive signaling (e.g., NF-κB), respectively. These signals promote a synergistic detoxification mechanism that protects against both hypoxia-induced ROS and chemotherapeutic agents [123]. ROS can also promote therapeutic resistance and disease progression through both oxidative DNA damage and functional suppression of tumor suppressors (e.g., p53, PTEN, and RB) [124]. Collectively, these findings highlight oxidative stress pathways as context-dependent regulators of both cytotoxicity and survival signaling, supporting ongoing interest in therapeutically targeting redox vulnerabilities in advanced PCa.

Like mitochondrial ROS, nitric oxide (NO) is reported to stabilize HIF-1α [125]. NO is generated during the conversion of L-arginine to L-citrulline via nitric oxide synthase (NOS), an oxygen-dependent enzymatic process, indicating that oxygen availability can directly influence NO production. Notably, the role of NO in PCa progression appears to be highly concentration-dependent. Lower NO levels are generally associated with tumor-promoting effects, whereas higher NO levels exert protective or anti-tumor activity [126]. In support of a chemosensitizing role for NO signaling, one study demonstrated that acute hypoxia (1% O_2_ for 12 h) reduced NO production in triple-negative breast cancer (MDA-MB-231) and mouse melanoma (B16F10) cells, which was associated with the development of doxorubicin resistance [127]. In PCa models, restoration of NO signaling re-sensitized PC3 cells to doxorubicin in hypoxia (0.5% O_2_ for 24 h), implicating NO and NO–HIF pathway crosstalk as potential targets for chemosensitization [128]. Collectively, these findings propose that hypoxia-dependent suppression of both ROS- and NO-associated cytotoxic signaling can diminish responses to genotoxic chemotherapy, highlighting redox and NO signaling pathways as potential therapeutic avenues for chemosensitization.

#### 3.1.3. Lipid Metabolism

Lipid metabolism has emerged as a potent mechanism of chemoresistance across multiple cancer types, including PCa [129,130]. Lipid droplets (LDs) are increasingly recognized as active organelles in cancer metabolism, with multifactorial roles in promoting membrane biogenesis, autophagy, and protection from cellular stressors, including ROS and lipid peroxidation (Reviewed in [131]). In accordance with this, CRPC cell lines (PC3, DU145) that survived 72 h of chemotherapy exposure (docetaxel, cisplatin, or etoposide) exhibited increased LD accumulation, and clinical datasets further revealed that elevated expression of LD-associated genes (e.g., PLINs, DGATs) correlated with poorer survival outcomes [130]. Pharmacologic inhibition of DGAT reduced LD abundance and sensitized cells to chemotherapy, supporting a model in which LDs promote drug tolerance by buffering toxic lipid species and limiting lipid peroxidation [130]. Importantly, suppression of lipid peroxidation can enhance survival by preventing ferroptosis, an iron-dependent form of regulated cell death driven by the accumulation of lipid peroxides [129].

Hypoxia appears to further reinforce these lipid-dependent survival programs [129,132]. Our group recently demonstrated that conditioning a CRPC cell line panel in acute hypoxia (1% O_2_ for 8 h) altered sensitivity to ferroptosis inducers relative to normoxia. Integrative transcriptomic and lipidomic analyses further revealed that hypoxia favored lipid profiles with reduced polyunsaturated fatty acid (PUFA) content, thereby decreasing susceptibility to lipid peroxidation. Consistent with this model, hypoxia-associated LD accumulation was proposed to sequester peroxidation-prone lipids and promote resistance to ferroptosis [129]. Notably, a similar shift in lipid profiles has been linked to aggressive phenotypes in PCa, including a 2015 report demonstrating that lipid-containing extracellular vesicles enhance PCa invasiveness [132].

Beyond lipid remodeling in the tumor cell, adipocyte-rich microenvironments may further contribute to metastatic progression and therapy resistance. Increased adipocyte populations within the prostate TME are associated with enhanced EMT, metastatic behavior, and chemoresistance [132,133]. Supporting the role of adipose-associated hypoxia in this process, clinical analyses have reported that periprostatic adipose tissue exhibits a hypoxia-associated transcriptional signature, alongside increased inflammatory and fibrotic markers [134]. These studies highlight lipid metabolism as a central and hypoxia-reinforced axis of therapeutic resistance in PCa and suggest that targeting lipid remodeling pathways and/or adipose–tumor interactions may represent a promising therapeutic strategy.

Collectively, these studies highlight several prevalent mechanisms of hypoxia-associated chemoresistance in PCa that also represent actionable therapeutic vulnerabilities, such as redox adaptation, mitochondrial maintenance, and lipid remodeling. However, it is important to acknowledge that additional resistance programs contribute to chemotherapy failure, including altered drug influx/efflux and metabolism, epigenetic reprogramming, non-coding RNAs, stromal- and immune-mediated survival signaling, and lineage plasticity [135,136,137,138]. Therefore, continued efforts to define how the duration of hypoxic exposure shapes chemoresistance will be critical for optimizing treatment paradigms and improving patient outcomes.

### 3.2. Targeted Therapies

As outlined throughout this review, both hypoxia-dependent and -independent activation of survival pathways and TME modulation contribute to multimodal therapeutic resistance in PCa. These challenges have driven increasing interest in targeted therapeutic strategies, including inhibitors of key signaling pathways (e.g., PIM Kinase, PI3K, PARP) and immune checkpoint inhibitors (CTLA4, PD-1/PD-L1).

#### 3.2.1. PI3K Pathway Inhibitors (PI3K/AKT/mTOR)

Hyperactivity of phosphatidylinositol-3-kinase (PI3K) is common in cancer due to dysregulated upstream signaling or genomic alterations, promoting tumor progression, metastasis, and therapeutic resistance [139,140]. Under hypoxia and other stressors, the PI3K/AKT/mTOR axis promotes cell survival through regulation of autophagy, proliferation, cell motility, and anti-apoptotic signaling [139,141]. In PCa, loss of the tumor suppressor PTEN results in constitutive PI3K activation and promotes castrate-resistance via reciprocal AR/PI3K crosstalk [56,79,89]). Subsequently, inhibitors targeting PI3K, AKT, and mTOR, alone or in combination, have been extensively evaluated in PCa models [142].

Despite its tumor-promoting role, targeted PI3K inhibition in PCa has proven challenging. In CRPC, resistance to PI3K inhibitors within the hypoxic bone TME has been linked to integrin α6β1 (ITGα6), an AR-regulated laminin receptor that promotes metastasis and survival [143,144]. Notably, ITGα6 inhibition sensitized CRPC cells (C4-2) to PI3K blockade [99]. Further investigation revealed that hypoxia-induced PIM kinase signaling protects against PI3K inhibition through Nrf2-mediated suppression of ROS. Dual PI3K targeting with PIM or ITGα6–PIM inhibitors sensitized cells in normoxia and hypoxia (1% O_2_ for 24–72 h) [99]. Building on these findings, multi-pathway inhibitors have shown increased potency. However, off-target gene activation has raised concerns regarding the toxicity associated with pan-kinase inhibition [145].

Dysregulation of the PI3K/AKT/mTOR signaling axis promotes PCa progression and therapeutic resistance, emphasizing PI3K as a compelling therapeutic target. However, the clinical success of PI3K inhibitors in PCa has been limited due to resistance and poor tolerability [146]. These challenges likely reflect tissue-specific PI3K isoform expression, extensive survival pathway crosstalk, and diverse TME stressors, such as nutrient availability and hypoxia, that buffer PI3K inhibition [146]. Collectively, these observations highlight the need for improved biomarkers and stratification strategies to identify patient cohorts most likely to benefit from PI3K targeted therapies.

#### 3.2.2. PIM Kinase Inhibitors

The *Proviral Integration site* for Moloney murine leukemia virus 1 (PIM1) kinase is a member of a family of oncogenic Serine/Threonine kinases that have been shown to promote therapeutic resistance. PIM1 levels are elevated in high-grade prostatic intraepithelial neoplasia (HG-PIN) relative to normal tissue and further elevated in PCa [147,148]. PIM1 promotes tumor progression through various mechanisms, impacting cell cycle progression, proliferation, and survival [96,98,147,148]. PIM is a particularly promising target to oppose tumor hypoxia because it is activated in response to hypoxia, independent of HIF-1 [149]. The most well-described role for PIM kinases in the context of cancer is promoting survival and escape from apoptosis. PIM directly phosphorylates and inactivates pro-apoptotic proteins (i.e., Bad and ASK1) [97,150]. In addition, growing evidence suggests that the ability of PIM to evade chemotherapy-induced apoptosis is, in part, because PIM suppresses oxidative stress through multiple angles. In PCa, PIM reduces the generation of ROS by increasing the antioxidant capacity of the cell (via activation of NRF2) [151]. These functions of PIM cause resistance to chemotherapies that rely on ROS-induced cell death. PIM1 expression also affects sensitivity to chemotherapies by increasing the activity of ATP-binding cassette (ABC) drug transporters and enhancing drug efflux [152]. Finally, PIM1 is also implicated in resistance to PI3K inhibitors, HT, and immunotherapy [76,99,153].

Given the extensive role of PIM in cancer survival and therapeutic resistance, several PIM inhibitors have been evaluated in clinical trials for PCa and other malignancies [154,155]. Despite promising pre-clinical data, clinical application of these inhibitors is limited due to efficacy and toxicity-associated challenges. A recent study revealed that inhibiting PIM catalytic activity paradoxically stabilizes all PIM isoform protein levels, subsequently promoting resistance to both catalytic PIM inhibitors and other therapies [154]. Interestingly, this same phenomenon was observed using a kinase-dead mutant, suggesting there are kinase-independent mechanisms of PIM. Excitingly, it was further demonstrated that targeted degradation of PIM using a proteolytic targeting chimera (PROTAC) was more cytotoxic in PCa models compared to catalytic PIM inhibitors. Thus, targeted degradation of PIM kinases remains a promising strategy to suppress oncogenic signaling in PCa [154].

#### 3.2.3. PARP Inhibitors

Poly (ADP-ribose) polymerase (PARP) is a family of 18 proteins that catalyze the transfer of ADP-ribose monomers or polymers to acceptor proteins in a process termed PARylation. Most PARP substrates engage in DNA synthesis and repair. Mechanistically, PARP inhibitors induce cell death by preventing homologous recombination (HR) repair. In brief, HR repair is initiated when the MRN complex senses double-strand breaks (DSBs) and activates ATM. BRCA1 then promotes DNA end resection to generate RPA-coated single-stranded DNA that engages ATR signaling. BRCA2 facilitates RAD51 loading to drive homology search and strand invasion, followed by repair completion through Holliday junction processing [156]. The redundancy of DNA repair pathways reduces the efficacy of PARP inhibitors, and as such, these drugs are most effective in treating cancers that harbor deficiencies in HR pathways responsible for DSB repair [157]. In these cancers, PARP inhibition promotes the accumulation of SSBs that are converted into DSBs during transcription, driving cell death through error-prone repair pathways, such as non-homologous end joining (NHEJ) [158,159].

PCa patients with metastatic disease more frequently harbor mutations in DNA repair-related genes (e.g., BRCA1/2) than those with localized disease [160]. Individuals with DNA repair mutations are ideal candidates for treatment with PARP inhibitors for the potential to exploit synthetic lethality. Synthetic lethality describes the presence of two genetic alterations, whether arising naturally or induced pharmacologically, that synergistically result in a loss of viability [161]. PARP inhibitors have been applied to PCa as both a monotherapy (e.g., olaparib, rucaparib, niraparib, and talazoparib) and as an adjuvant treatment to hormone therapy, immunotherapy, chemotherapy, targeted therapies, and radiation [158,162]. Across cancer types, >40% of BRCA-deficient cancer patients fail to respond to PARP inhibitors, and most eventually acquire resistance to oral inhibitors [163,164]. Major mechanisms of resistance to PARP inhibitors in HR-deficient cancers include reversion mutations in BRCA-mutated mCRPC, protection of the DNA fork during replication, epigenetic modifications, restoration of PARylation, and re-emergence of the HR pathway [163,164].

The study of hypoxia’s role in resistance to PARP inhibitors has largely focused on the impact of oxygen tension and exposure length on HR repair, as both characteristics differentially affect related pathways [165]. Evidence suggests that severe acute hypoxia (we define as ≤0.5% O_2_ for ≤24 h) upregulates HR repair by altering activation and signaling of related proteins (e.g., ATM/ATR kinases [166], RRM1/2 [167], and SETX [168]. Conversely, severe prolonged hypoxic exposure (we define as ≤0.5% O_2_ for ≥48 h) reduces the expression and activity of HR repair genes and displays synthetic lethality with PARP inhibition [165,169,170,171]. This effect is lost with increased oxygen tension, as one study found upon investigating the expression of repair genes after extended exposure to hypoxia (48 h) in breast cancer cell lines (MDA231). In 0.5% O_2_, BRCA1 and RAD51 mRNA levels were significantly suppressed, and cells were sensitized to PARP inhibitors. Conversely, cells cultured in 2% O_2_ experienced increased resistance to PARP inhibitors in both HR-proficient and -deficient models compared to normoxia [169]. These studies suggest that short-term exposures to hypoxia promote resistance to PARP inhibition, whereas prolonged exposure to severe hypoxia reduces DNA repair activity, potentially enhancing drug efficacy. Interestingly, cyclic anoxia (0% O_2_ 30 min–3 h) has not been found to alter RAD51 expression or sensitivity to PARP inhibitors in non-small cell lung cancer (H1299) and cervical epidermoid carcinoma lines (ME180), supporting that efficacy is limited to more chronically hypoxic tumor regions [172]. Nevertheless, further investigation into the relationship between oxygen tension and exposure duration on PARP inhibitor efficacy is warranted.

The potential to improve drug efficacy by leveraging tumor hypoxia has been validated in vivo. In a xenograft model of human cervical cancer (IGROV1), combined treatment with olaparib and the anti-angiogenic agent, cediranib, caused a downregulation of HR proteins, BRCA1/2 and RAD51, independent of tumor hypoxia. Cediranib treatment increased tumor hypoxia, and HR proteins were significantly reduced in hypoxic tumor cells compared to their normoxic counterparts, highlighting hypoxia as a secondary mechanism increasing drug efficacy [173]. These findings were validated in a clinical trial, which showed that mCRPC patients who received a combination of cediranib and olaparib experienced a significant increase in radiographic progression-free survival compared to olaparib alone (8.5 and 4.0 months, respectively). However, combination therapy was associated with greater toxicity than PARP inhibition alone, highlighting the potential value of developing prodrugs responsive to defined oxygen tensions and exposure durations, or anti-angiogenic agents capable of uniformly inducing hypoxia throughout a tumor [174].

#### 3.2.4. Immunotherapy

Immunotherapy is a cornerstone treatment for several malignancies, most notably through immune checkpoint inhibitors (ICIs) targeting the PD-1/PD-L1 axis (e.g., Pembrolizumab, Atezolizumab) and CTLA-4 (e.g., Ipilimumab). Excitingly, these approaches have demonstrated significant clinical benefit for several cancers, including lung, head and neck, cervical, and metastatic melanoma [175]. Mechanistically, these therapies disrupt inhibitory checkpoint signaling that restrains antitumor T cell activity, thereby enabling cytotoxic T cell–mediated tumor cell killing. However, clinical responses to ICIs are highly context dependent and require a TME that supports immune infiltration, antigen presentation, and sustained effector function [176].

ICIs exhibit limited efficacy in so-called “immune-cold” TMEs, which are broadly characterized by reduced antitumor immune cell populations (e.g., CD8^+^/CD4^+^ T lymphocytes, natural killer cells, dendritic cells), increased immunosuppressive populations (e.g., regulatory T cells, M2-like macrophages, myeloid-derived suppressor cells), dense stromal architecture, and elevated expression of immunosuppressive markers [176]. Hypoxia is a well-established driver of immune suppression and immune exclusion, in part through nutrient depletion, extracellular acidification, and stromal remodeling that collectively shape hypoxic TMEs. Moreover, hypoxia promotes increased secretion of metabolites, cytokines, and chemokines that favor expansion of immunosuppressive cells over anti-tumor immune cells, including lactate, TGF-β, and CXCR4/CXCL12 [55,177,178,179].

Consistent with these observations, immune checkpoint blockades have historically demonstrated limited efficacy in PCa [180,181]. In 2018, a pivotal study demonstrated that hypoxic prostate tumors exhibited minimal T lymphocyte infiltration and increased resistance to both PD-1 and CTLA-4 ICIs. Notably, these tumors could be sensitized to ICIs when paired with a hypoxia-activated prodrug [180]. Additionally, high PIM1 expression is associated with hypoxia and immunosuppressive tumor environments [149,153]. Although not evaluated directly in the context of hypoxia, PIM inhibition was shown to enhance sensitivity to anti–PD-1 therapy in syngeneic and xenograft mouse models of PCa [153]. HIF-1α is also a potential target for improving immunotherapy efficacy. Beyond driving metabolic adaptations that reshape the TME, HIF-1α can limit cytotoxic T cell activity by modulating PD-L1 and MHC class I expression and reducing macrophage phagocytosis by downregulating CD47 expression [181]. In support of this concept, recent studies demonstrated that HIF-1α inhibition improves the efficacy of PD-1 blockade and is associated with a shift toward increased cytotoxic T cell representation and reduced suppressive immune cell populations [182].

Together, these findings highlight the multidimensional role of hypoxia in limiting immunotherapy efficacy in PCa and reinforce the need for therapeutic strategies that target hypoxic tumor regions to enable immune engagement. Despite hypoxia’s well-established immunosuppressive role, there are limited studies that directly investigate the interplay of temporal hypoxic subtypes and ICIs, likely due, in part, to the poor clinical efficacy of ICIs in PCa. Nevertheless, improving the efficacy of ICIs in PCa and other immune-cold solid tumors remains an important and active area of investigation. Emerging approaches aimed at microenvironmental remodeling, including targeting immunosuppressive metabolites such as lactate, have shown promising results in preclinical models [183,184]. Consideration of hypoxic influence on microenvironmental changes would be beneficial to elucidate the impact of temporal subtypes on ICI efficacy and may also reveal additional sensitization strategies.

#### 3.2.5. HIF Inhibitors/Hypoxia-Activated Prodrugs

Given that HIF signaling is a central driver of hypoxic adaptation, HIF-1 has been extensively pursued as a therapeutic target, supported by strong preclinical evidence in PCa [183,184] and other malignancies [185]. However, these approaches have not translated broadly due to the difficulty in targeting transcription factors, the transient nature of HIF-1 activation, and limited therapeutic windows due to the essential role of HIF signaling in normal physiological response to oxygen fluctuations [185,186,187]. Pharmacologically, transcription factors also remain difficult to drug directly, although emerging approaches in drug design (e.g., molecular glues) and advances in delivery formulations (e.g., nanomedicine) may improve efficacy [154,188]. Importantly, most studies do not distinguish acute from chronic hypoxia when evaluating HIF-1 inhibitors. Investigating HIF-1 inhibition in more diverse hypoxic conditions would clarify their therapeutic efficacy beyond disruption of early or acute hypoxia responses [115]. Notably, HIF-2 specific inhibitors have demonstrated clinical promise in patients with VHL disease and renal cell carcinoma [189]. Belzutifan (MK-6482, previously called PT2977) is FDA-approved for the treatment of VHL disease patients [185]. HIF-2 inhibitors have shown several common treatment adverse effects, including anemia and respiratory failure. To date, HIF inhibitors have not been widely successful outside of VHL disease, and only a subset of renal cancer patients have reported partial responses. However, their clinical utility in PCa has yet to be evaluated.

Beyond HIF inhibition, hypoxia-activated prodrugs (HAPs) have shown preclinical promise by selectively targeting hypoxic tumor regions and enhancing sensitivity to standard therapies, although challenges in drug delivery, efficacy, and toxicity have also limited clinical success [190,191]. The most advanced example in this class is TH-302 (Evofosfamide), a second-generation HAP that consists of a 2-nitroimidazole moiety linked to a DNA cross-linking agent. Under hypoxic conditions, the 2-nitroimidazole is reduced and the cytotoxic cargo is released [186]. Notably, Cytochrome P450 is required for this reduction reaction, which limits the scope of tumors that could potentially respond to this drug [187]. TH-302 has shown promise in preclinical studies of many solid tumor types, particularly in combination with chemotherapy and anti-angiogenic agents. Notably, TH-302 has been investigated in clinical trials for over a decade, but it has not progressed further due to a lack of efficacy and adverse events, such as skin and/or mucosal toxicity, thrombocytopenia, neutropenia, and myelosuppression [188]. Together, these findings suggest that an investigation into alternative approaches to target hypoxia is needed.

## 4. Conclusions

PCa heterogeneity reflects the combined influence of genomic alterations, tumor cell plasticity, and selective pressures imposed by therapy and the TME. Hypoxia plays a complex and multidimensional role in shaping the behavior of localized and metastatic PCa by driving therapy resistance through promotion of compensatory survival programs, including AR signaling, DNA damage responses, metabolic rewiring, and immune suppression to support tumor persistence.

Tissue hypoxia is spatially and temporally dynamic, and tumor cell responses depend on both the severity and duration of oxygen limitation. However, the duration of hypoxic exposures that define acute, cyclic, and chronic hypoxia varies widely across studies [124,189]. While acute hypoxia has been extensively studied, both chronic and cyclic hypoxia remain understudied despite their evident clinical relevance [19,20,190,191,192]. Chronic hypoxia imposes persistent selective pressure through extensive oxygen deprivation that drives resilient and aggressive tumor cell phenotypes. These characteristics include enhanced stemness, genomic instability, and metabolic adaptation that promote metastatic potential and therapeutic resistance [124,193,194]. In cyclic hypoxia, the continuous hypoxia-reoxygenation cycles impose recurrent oxidative stress that necessitates enhanced stress-response and survival mechanisms. These fluctuations may also select for cell populations with high metastatic potential [19,124,191,192]. Despite these observations, very few studies have directly examined the epigenetic, transcriptional, and post-translational changes that are required to adapt to chronic hypoxia, or how they compare to those induced by cyclic hypoxia [19,20,193]. Consequently, it is unclear if the characteristics acquired under chronic hypoxia impart distinct advantages relative to metastatic potential and/or therapeutic resistance, or if there is substantial overlap with cyclic hypoxia. This ambiguity can be attributed in part to variation in experimental design imposed by differing model systems, hypoxic exposure frequency, duration, and oxygen tension [19,189]. As a result, the term hypoxia is used globally to describe a multitude of conditions that have obscured different biological outcomes from acute, cyclic, and chronic oxygen deprivation. While defining parameters for each hypoxia subtype is necessary for conceptual clarity, it is complex and context-dependent. Therefore, a baseline set of definitions that can be adapted across biological systems may provide a valuable approach for improved interpretability.

Translating these conceptual distinctions into clinically meaningful categories will be essential for improving patient stratification and limiting therapeutic resistance. Hypoxia remains difficult to measure in vivo due to its spatial heterogeneity and temporal dynamics, and current imaging and biopsy-based approaches often capture only a static or indirect view of tumor oxygenation [195,196]. Histologically, hypoxia is most often inferred using surrogate markers such as HIF-1α or expression of downstream targets (e.g., GLUT1 and CAIX), but they do not resolve the duration or history of hypoxic exposure. Moreover, they have also been reported as independent prognostic markers, irrespective of hypoxia, in some contexts [196,197]. Further research is needed to develop accurate biomarkers to distinguish between acute, chronic, and cyclic hypoxia exposure, which would enable a more informed approach to hypoxia-targeted therapies that have thus far been limited by toxicity, narrow therapeutic windows, and incomplete consideration of hypoxic duration.

Taken together, these challenges reinforce the need to refine hypoxia subtype definitions, improve clinical assessment of tumor oxygenation, and develop innovative strategies to overcome hypoxia-driven therapeutic resistance. Ultimately, the central challenge is that hypoxia is more dynamic than binary. Thus, therapeutic success may require either temporally matched interventions or targeting downstream dependencies that persist across hypoxia states. Integrating temporal multi-omic analyses may help resolve hypoxia-associated adaptations and identify biomarkers that better capture hypoxic history in patients. In parallel, adopting more physiologically relevant preclinical systems, such as patient-derived organoids, may improve our ability to model prolonged hypoxic exposure and uncover therapeutic opportunities with clearer clinical relevance.

## Data Availability

No new data was created or analyzed in this study. Data sharing is not applicable to this article.

## Abbreviations

ADT: androgen deprivation therapy; AR: androgen receptor; BCR: biochemical recurrence; BOLD: blood oxygen level-dependent; CRPC: castration-resistant prostate cancer; CSPC: castration-sensitive prostate cancer; DSBs: double-strand breaks; HIF: hypoxia-inducible factor; HR: homologous recombination; HRE: hypoxia response elements; ICI: immune checkpoint inhibitor; LD: lipid droplet; MRI: magnetic resonance imaging; NO: nitric oxide; PARP: Poly(ADP-ribose) polymerase; PCa: prostate cancer; PSA: prostate-specific antigen; ROS: reactive oxygen species; RP: radical prostatectomy; SSB: single-strand breaks; TME: tumor microenvironment; VHL: von Hippel–Lindau.
